# The effects of exogenous growth factors on matrix metalloproteinase secretion by human brain tumour cells

**DOI:** 10.1054/bjoc.1999.0876

**Published:** 1999-12-08

**Authors:** H K Rooprai, G J Rucklidge, C Panou, G J Pilkington

**Affiliations:** Experimental Neuro-oncology Group, Department of Neuropathology, Institute of Psychiatry, De Crespigny Park, London, SE5 8AF, UK; Department of Biochemistry, The Rowett Research Institute, Greenburn Road, Bucksburn, Aberdeen, AB2 9SB, UK

**Keywords:** growth factors, matrix metalloproteinases, brain tumour invasion

## Abstract

Matrix metalloproteinases (MMPs) are a growing family of zinc-dependent endopeptidases that are capable of degrading various components of the extracellular matrix. These enzymes have been implicated in a variety of physiological and pathological conditions including embryogenesis and tumour invasion. The synthesis of many MMPs is thought to be regulated by growth factors, cytokines and hormones. In this study, we investigated the effects of five exogenous growth factors known to be expressed by gliomas [epidermal growth factor (EGF), basic growth factor (bFGF), transforming growth factor beta (TGF-β1,2) and vascular endothelial growth factor (VEGF)] on MMP-2 and MMP-9 expression in an ependymoma, two grade III astrocytomas, a grade III oligoastrocytoma and a benign meningioma. Zymogram analysis revealed that the effects of the growth factors depended upon the cell lines used in the study. Growth factors generally up-regulated MMP-2 and MMP-9 expression in the gliomas but were least effective in the meningioma; the effect being most prominent with TGF-β1 and TGF-β2 in all the cell lines. It is hypothesized that paracrine growth factor interplay may be crucial in the regulation of MMP expression by glioma invasion of the normal brain. © 2000 Cancer Research Campaign


*British Journal of Cancer* (2000) **82**(1), 52–55
© 2000 Cancer Research Campaign
Article no. bjoc.1999.0876

### The effects of exogenous growth factors on matrix metalloproteinase secretion by human brain tumour cells

**HK Rooprai[1], GJ Rucklidge[2], C Panou[1] and GJ Pilkington[1]**

[1]Experimental Neuro-oncology Group, Department of Neuropathology, Institute of Psychiatry, De Crespigny Park, London SE5 8AF, UK; [2]Department of Biochemistry, The Rowett Research Institute, Greenburn Road, Bucksburn, Aberdeen AB2 9SB, UK

**Summary** Matrix metalloproteinases (MMPs) are a growing family of zinc-dependent endopeptidases that are capable of degrading various components of the extracellular matrix. These enzymes have been implicated in a variety of physiological and pathological conditions including embryogenesis and tumour invasion. The synthesis of many MMPs is thought to be regulated by growth factors, cytokines and hormones. In this study, we investigated the effects of five exogenous growth factors known to be expressed by gliomas [epidermal growth factor (EGF), basic growth factor (bFGF), transforming growth factor beta (TGF-β1,2) and vascular endothelial growth factor (VEGF)] on MMP-2 and MMP-9 expression in an ependymoma, two grade III astrocytomas, a grade III oligoastrocytoma and a benign meningioma. Zymogram analysis revealed that the effects of the growth factors depended upon the cell lines used in the study. Growth factors generally up-regulated MMP-2 and MMP-9 expression in the gliomas but were least effective in the meningioma; the effect being most prominent with TGF-β1 and TGF-β2 in all the cell lines. It is hypothesized that paracrine growth factor interplay may be crucial in the regulation of MMP expression by glioma invasion of the normal brain. © 2000 Cancer Research Campaign

**Keywords:** growth factors; matrix metalloproteinases; brain tumour invasion

The most common type of primary intracranial tumours in man are gliomas (Russell and Rubenstein, 1989). These tumours are characterized by their high invasive potential and display a wide diversity of histological appearances. Even low-grade gliomas infiltrate the contiguous brain; a key feature which precludes their successful therapy.

The underlying molecular mechanisms of brain tumour invasiveness are complex and involve a series of sequential steps, including modification of adhesive properties of tumour cells, degradation and remodelling of extracellular matrix (ECM) components by proteases such as matrix metalloproteinases (MMPs or matrixins) and migration of the tumour cells into the modified region (Liotta, 1986). It is possible that each of these steps may be modulated by a variety of interactive factors (Yamada, 1983).

MMPs are a superfamily of zinc-dependent endopeptidases which cooperatively degrade all components of the ECM. There are now at least 17 structurally related members in this family which are either secreted or membrane-bound. The family includes five subclasses: collagenases, gelatinases, stromelysins, metalloelastase and membrane-type metalloproteinases (MT-MMPs). MMPs have been implicated in various physiological and pathological conditions such as normal embryogenesis and tissue morphogenesis as well as tumour cell invasion and metastasis (Woessner, 1991; Romanic and Madri, 1994). Emerging literature suggests that brain tumour cell invasion utilizes numerous proteases in vitro, of which the predominant ones may be gelatinase-A and -B (MMP-2 and -9 respectively) (Rooprai and McCormick, 1997). As might be expected of enzymes with such degradative potential, the final in vivo level of activity of MMPs is tightly controlled and is thought to result from the interactions of many factors at different levels. It is well documented that the regulation of MMP expression is complex. The synthesis of many MMPs and their inhibitors, tissue inhibitors of metalloproteinases (TIMPs) is thought to be regulated by growth factors, cytokines and hormones.

There is a vast amount of data in vivo which suggests that stromal cells of other tumours, probably stimulated by growth factors produced by malignant cells are the sole producers of MMPs, even when malignant cells are positive for MMPs at the protein level (McDougall and Matrisian, 1995). Indeed, a proposed explanation for the differences between in vitro and in situ levels of MMPs in gliomas (Reubi et al, 1989) is that local microenvironmental conditions in vitro stimulate such MMP activity while in vivo these enzymes are produced locally and on demand in very small quantities (Rooprai and McCormick, 1997). Similarly, the cell type of origin of MMP expression in gliomas is probably not only endothelial (known to be migratory during embryogenesis) but also neoplastic glia. It is debatable whether endothelial-derived or neoplastic glial-derived MMPs are used in the diffuse invasive behaviour of gliomas.

Growth factors are mitogens and exert their effects by interacting with particular cell surface receptors. They can also control migration and invasion. Growth factors such as epidermal growth factor (EGF), basic fibroblast growth factor (bFGF), platelet-derived growth factor (PDGF) and transforming growth factor beta (TGF-β) have all been shown to stimulate human glioma cell invasion (Finn et al, 1997; Laerum, 1998).

*Received 14 April 1999*
*Revised 25 June 1999*
*Accepted 30 June 1999*

*Correspondence to:* HK Rooprai

**Table 1** Cumulative results for MMP-2 and -9 expression in five cell lines under the influence of five exogenous growth factors in vitro

| Growth factors | Control | | EGF | | bFGF | | TGF-β1 | | TGF-β2 | | VEGF | |
|---|---|---|---|---|---|---|---|---|---|---|---|---|
| MMPs | 2 | 9 | 2 | 9 | 2 | 9 | 2 | 9 | 2 | 9 | 2 | 9 |
| IPRPE-M | P | P | – | – | – | – | + | – | + | – | – | – |
| IPMA-E | P | P | + DB | – | + DB | ++ | + | ++ | + | ++ | – | + |
| IPMC-A3 | P (DB) | P | + DB | ++ | + DB | ++ | ++DB | + | +++DB | + | +DB | + |
| IPHAB-A3 | P (DB) | P | + DB | ++ | + DB | + | + DB | ++ | + DB | ++ | + DB | ++ |
| IPAB-AO3 | P | A | ++DB | ++ | ++DB | + | +++DB | + | ++ | + | ++DB | + |

P = Presence of enzyme, A = absence of enzyme, DB = doublet (both active and latent forms of MMP present); + = up-regulation of MMP secretion, – = down-regulation of MMP secretion. Growth factors: EGF = epidermal growth factor, bFGF = basic fibroblast growth factor, TGFβ = transforming growth factor, VEGF = vascular endothelial growth factor. Cell lines: IPRPE-M = meningioma, IPMA-E = ependymoma, IPMC-A3 and IPHAB-A3 = Grade 3 astrocytomas, IPAB-AO3 = Grade 3 oligoastrocytoma.

We have previously shown that there were discrepancies in MMP expression in a number of gliomas of various histological grade and malignancy, when comparative analysis was performed with immunohistochemistry, immunocytochemistry and zymography (Rooprai et al, 1998). Although zymography results showed that MMP-2 was predominantly expressed, corresponding paraffin sections showed no expression of either MMP-2, -9 or -14 within tumours. We proposed that these differences may be explained by the proliferative state of the cells in vitro, the passage number of the cell lines, presence of various growth factors, gangliosides and cell adhesion molecules, levels of TIMPs, genetic changes of glioma cells in vitro and the cellular heterogeneity of gliomas in vivo. Two of these factors, the influence of gangliosides (Maidment et al, 1996) and passage number (Rossi et al, 1996) have been investigated experimentally by our group. The aim of this study was to assess whether any of a panel of growth factors known to be expressed by brain tumours influence production of MMPs by glioma and meningioma cells in vitro.

### MATERIALS AND METHODS

### Cell cultures

Surgical samples from patients with various types of brain tumours were obtained from the neurosurgical staff at Kings' College Hospital, London. Biopsy material was used to establish five short-term cultures at low passages. All the samples analysed were diagnosed by a neuropathologist, according to the World Health Organization criteria (Kleihues et al, 1993). The samples included one meningioma, one ependymoma, two astrocytomas (grade III) and one oligoastrocytoma (grade III).

Cells were cultured as monolayers in small plastic culture flasks (Marathon) at 37°C, 5% carbon dioxide in a standard humidified incubator. All the cell lines used in this study were at low passages (less than P5). Cells were initially maintained in Dulbecco's modified Eagle's medium (DMEM) supplemented with antibiotic–antimycotic (at a final concentration of 100 IU penicillin, 100 μg streptomycin and 0.25 μg of amphotericin per ml) and 10% fetal calf serum (Sigma). This medium was discarded as the monolayers attained 70% confluence and replaced on sequential days with media supplemented with 7%, 3% and finally serum-free media containing one of the following growth factors at 10 ng $ml^{-1}$: EGF, bFGF, TGF-β1 and TGF-β2 and vascular endothelial growth factor (VEGF). The working concentration of 10 ng $ml^{-1}$ was chosen as our group has previously shown this to be optimum for glioma invasion in vitro (Merzak et al, 1994). All growth factors were obtained from Sigma as lyophilized powders and reconstituted in DMEM to give stock solutions of 100 μg $ml^{-1}$. The cells were incubated with growth factors under serum-free conditions for 48 h, the medium was harvested, dialysed against water for 16 h individually and freeze-dried. The original volumes of these cell-conditioned medium samples, which served as the source of matrix metalloproteinases for zymography, were noted.

### Zymography

Zymogram analysis was performed by a modification of the methods of Heussen and Dowdle (1980) and Rucklidge et al (1990). Briefly, the substrate gelatin (with a final concentration of 1 mg $ml^{-1}$) was co-polymerized into an 11% acrylamide resolving gel at the time of gel casting. The freeze-dried samples were dissolved in Tris buffer containing 30% glycerol, 7.7% sodium dodecyl sulphate (SDS) and 0.3% bromophenol blue at pH 6.8 (without heating) and aliquots of media equivalent to a standardized number of cells (4800) were used. The samples were loaded into 5% stacking gel wells and electrophoresed in a Mini Protean II Dual Cell (Bio-Rad). The samples were not activated before running which allows the latent and active forms of each enzyme to be visualized. The gels were immersed in Triton X-100 (2.5% in water) for 3 h to deactivate the enzymes by removing the SDS. The enzymes were then reactivated by incubating the gels in a 50 mM Trizma–HCl buffer (pH 7.6) containing 10 mM calcium chloride ($CaCl_2$) at 37°C overnight. The gels were stained in 0.025% Coomassie blue in methanol:acetic acid:water (10:1:10 v:v:v) for 4 h and destained with methanol:acetic acid:water (2:1:10 v:v:v) for 24 h to reveal clear bands containing proteolytic activity on a dark blue background. The bands were identified in terms of their molecular weights: 92 kDa and 72 kDa, corresponding to MMP-9 and -2 respectively. Proteolytic activity was evaluated photodensitometrically using an IBAS 200 image analyser and verification of the MMP status of enzymes detected was achieved by substituting the 10 mM $CaCl_2$ with an MMP inhibitor, 1,10-phenanthroline.

### RESULTS

Table 1 represents cumulative results for the zymogram analysis of gelatinase-A and -B expression (MMP-2 and -9 respectively) in five cell lines derived from human brain tumour, under the influence of five exogenous growth factors. Each row represents a cell line and each column represents MMP-2 and MMP-9 expression in the presence of various growth factors, compared to the

*© 2000 Cancer Research Campaign*

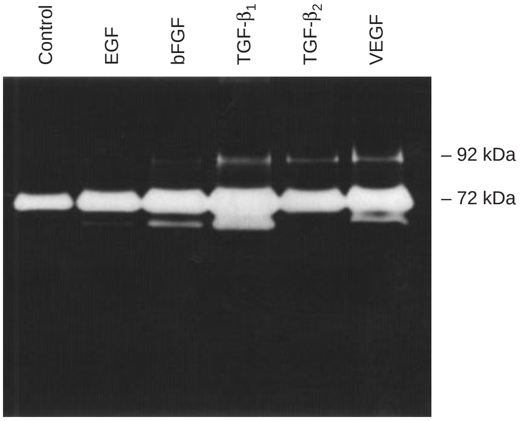

**Figure 1** A representative gel of a zymogram for the grade 3 oligoastrocytoma-derived cell line (IPAB-AO3). The intense 72 kDa gelatinase (MMP-2) appears as well-defined sets of bands underneath the less intense 92 kDa (MMP-9) bands. Both enzymes can be seen to be up-regulated by the various growth factors with respect to the controls. The doublet band configuration is indicative of the presence of both the activated and latent form of MMP-2. EGF = epidermal growth factor, bFGF = basic fibroblast growth factor, TGF-β = transforming growth factor beta, VEGF = vascular endothelial growth factor controls. The presence or absence of each enzyme in the controls is indicated by + and – respectively, whereas its up-regulation is indicated by + and down-regulation by –. The presence of doublets (DB) implies that the enzyme is present in both the active and latent forms. Figure 1 represents a typical gel obtained by zymogram analysis. The intensity of the clear bands on the dark background is proportional to the proteolytic activity. This is also indicated by ++ or +++ in the table.

The results showed that the effects of the exogenous growth factors depended upon the cell line used in the study. All the growth factors generally upregulated both MMP-2 and MMP-9 secretion in the ependymoma and three grade 3 astrocytomas while downregulation of MMP-2 and -9 was seen in the meningioma (IPRPE-M) under the influence of all the growth factors, with only two exceptions (Table 1). In the case of the ependymoma (IPMA-E), down-regulation was seen in MMP-9 with EGF and in MMP-2 with VEGF. Interestingly, the effects on up-regulation of MMP secretion were most prominent with TGF-β1 and TGF-β2 in all the cell lines studied.

### DISCUSSION

The results indicate that the exogenous growth factors investigated are capable of regulating the secretion of MMP-2 and -9 by human brain tumours in vitro. The most effective stimulators of the MMPs were TGF-β1 and TGF-β2. To date, there have been limited reports in the literature on the effects of exogenous growth factors on MMP expression. However, the findings in the present study substantiate previous work by other groups who have reported that TGF-β1 up-regulates these MMPs in cultured human karatinocytes (Salo et al, 1991) and human fibroblasts (Overall et al, 1991). The possibility that TGF-β1 can also up-regulate other MMPs in gliomas has previously been investigated by Nakano et al (1993) who reported that in two out of four glioma cell lines, this growth factor up-regulated matrilysin (MMP-7) expression. The present study also indicates that MMP-2 expression is up-regulated by VEGF, a finding which is consistent with that of Lamoreaux et al (1998) who reported that VEGF up-regulates MMP-2 (and down-regulates TIMPs-1 and -2) in microvascular endothelial cells. A very recent report (Tamaki et al, 1998) suggests that C6 astrocytoma cells appear to have a dual regulatory mechanism for MMP-2 expression which are dependent on cell density. At low density, these cells respond to bFGF and VEGF by up-regulating MMP-2 activity, whereas at high density MMP-2 is down-regulated by released ECM molecules in response to the two growth factors. The authors suggested that these regulatory mechanisms may play a role in the coordination of tumour-associated angiogenesis by malignant glial cells.

A number of previous studies have clearly shown that growth factors (EGF, bFGF, PDGF and TGF-β) stimulate human glioma cell invasion (Engebraaten et al, 1993; Lund-Johansen et al, 1990, 1992; Pedersen et al, 1994). In particular, TGF-β1 acts primarily as a growth inhibitor (Heldin and Westermark, 1989). Moreover, our group has shown that TGF-β1 has a dual effect on glioma cells. In one study, it had a growth inhibitory effect in vitro both on human fetal astrocytes and three cell lines derived from human gliomas of different histological type and grade of malignancy while it is strongly induced stimulation of migration and invasion by the same cell lines into Matrigel (Merzak et al, 1994). In another study, different concentrations and different combinations of growth factors gave varied responses with respect to proliferation and invasion of gliomas in vitro suggesting synergism–antagonism between growth factors. Indeed, both negative and positive effects of TGF-β1 on the mitogenic actions of EGF, PDGF and bFGF were seen on a pilocytic astrocytoma cell line (Merzak et al, 1995) while only a negative effect was reported on the mitogenic action of these other growth factors on malignant glioma cell lines, except when it was combined with bFGF (Merzak et al, 1994).

The mechanisms underlying stimulation of glioma invasion are not well understood. It is possible that growth factors may influence the invasion cascade via ganglioside-mediated ECM adhesion (Pilkington et al, 1993) and also promote invasion by controlling the balance of ECM protein synthesis and degradation (Koochekpour et al, 1995). Moreover, the present study indicates that TGF-β1 can up-regulate MMP-2 and -9 in gliomas, while earlier reports (Stetler-Stevenson, 1990) showed that tissue inhibitor of metalloproteinase 2 (TIMP-2) gene expression is down-regulated in human tumour cell lines by this factor. Moreover, all isoforms of TGF-β have also been shown to be abundant at the brain–tumour interface in 64 gliomas, using immunohistochemical techniques (Merzak et al, submitted). TGF-β1 was found to be most frequently and TGF-β3 the least frequently expressed. Both of these growth factors were located in the centre of the tumour in invasive cells and in endothelial cells. The three isoforms of TGF-β and the receptor TGF-β type II were very frequently co-expressed in the tumours suggesting that these factors may act synergistically in an autocrine manner to promote glioma progression (Merzak et al, submitted). Further studies include functional blocking by antibodies to various growth factors in a larger number of brain tumours as well as investigating the roles of TGF-β isoforms in the invasive and immunosuppressive behaviour of human gliomas in vitro. It is possible that up-regulation of MMP expression and invasion of the normal brain by neoplastic glia may result in response to paracrine growth factors similar to the mechanisms proposed by Nicholson et al (1994) who

*© 2000 Cancer Research Campaign*

investigated the effects of neutrophins and transferrins on degradative enzymes in malignant melanoma cells in the metastatic cascade.

The precise mode of action of these growth factors in modulating tumour invasion remains unclear, although growth factors have been shown to regulate MMP secretion, laminin and hyaluronic acid production, cell surface ganglioside expression and adhesion molecule function. It can be concluded that all growth factors used in this study induced MMP-2 and -9 secretion generally in the gliomas and ependymoma. The meningioma behaved differently, showing mainly down-regulation of the MMPs. One possible hypothesis for this observation is that meningiomas are not neuro-ectodermal in origin and therefore may be biologically different. The growth factor receptor status in meningiomas is also likely to be different from that in gliomas (Romanic and Madri, 1994). Alternatively, gliomas and meningiomas may not express the same type and levels of MMPs. Preliminary data from one study shows that malignant meningiomas have minimal expression of MMPs compared to atypical and benign ones (Rooprai et al, unpublished data). Local microenvironmental conditions in vitro may involve mechanisms which appear to stimulate such MMP activity in gliomas. It is also possible that paracrine growth factors interplay between heterogeneous glioma cells may play a role in the regulation of MMP secretion at the brain–tumour interface thereby facilitating invasion on neoplastic glial cell secreted ECM.

### ACKNOWLEDGEMENTS

This work was supported by the Association for International Cancer Research.

### REFERENCES

Engebraaten O, Bjerkvig R, Pedersen PH and Laerum OD (1993) Effects of EGF, bFGF, NGF and PDGF(bb) on cell proliferative, migratory and invasive capacities of human brain tumour biopsies in vitro. *Int J Cancer* **53**: 209–214

Finn PE, Bjerkvig R and Pilkington GJ (1997) The role of growth factors in the malignant and invasive progression of intrinsic brain tumours. *Anticancer Res* **17**: 4163–4172

Heldin C-H and Westermark B (1989) Growth factors as transforming proteins. *Eur J Biochem* **184**: 487–496

Heussen C and Dowdle EB (1980) Electrophoretic analysis of plasminogen activators in polyacrylamide gels containing sodium dodocyl sulfate and copolymerized substrates. *Anal Biochem* **102**: 196–202

Kleihues P, Burger C and Scheithauer BW (1993) Histological typing of tumours of the central nervous system. In: *WHO Classification*, 2nd edn. Springer Verlag: Berlin

Koochekpour S, Merzak A and Pilkington GJ (1995) Growth factors and gangliosides stimulate laminin production by human glioma cells in vitro. *Neurosci Lett* **186**: 53–56

Laerum OD (1998) The role of growth factors and oncogenes in tumour cell invasion. In: *Brain Tumour Invasion: Biological, Clinical and Therapeutic Considerations*, Mikkelsen, Bjerkvig R, Laerum OD and Rosenblum (eds), pp. 323–341

Lamoreaux WJ, Fitzgerald MEC, Reiner A, Hasty KA and Charles ST (1998) Vascular endothelial growth factor increases release of gelatinase A and decrease release of tissue inhibitors of metalloproteinases by microvascular endothelial cells in vitro. *Microvascular Res* **55**: 29–42

Liotta LA (1986) Tumor invasion and metastases: role of the extracellular matrix (Rhodes Memorial Award Lecture). *Cancer Res* **46**: 1–7

Lund-Johansen M, Bjerkvig R, Humphrey PA, Bigner SH, Bigner DD and Laerum OD (1990) Effect of epidermal growth factor on glioma cell growth, migration and invasion in vitro. *Cancer Res* **50**: 6039–6044

Lund-Johansen M, Forsberg K, Bjerkvig R and Laerum OD (1992) Effects of growth factors on human glioma cell line during invasion into rat brain aggregates in culture. *Acta Neuropath* **84**: 190–197

McDougall JR and Matrisian LM (1995) Contribution of tumour and stromal matrix metalloproteinases to tumour progression, invasion and metastasis. *Cancer and Metastas Rev* **14**: 351–362

Maidment SL, Merzak A, Koochekpour S, Rooprai HK, Rucklidge GJ and Pilkington GJ (1996) The effects of exogenous gangliosides on matrix metalloproteinase secretion by human glioma cells in vitro. *Eur J Cancer* **32**: 868–871

Merzak A, McCrea S, Koochekpour S and Pilkington GJ (1994) Control of human glioma cell growth, migration and invasion in vitro by transforming growth factor beta-1. *Br J Cancer* **70**: 199–203

Merzak A, Koochekpour S, Dkhissi F, Raynal S, Lawrence D and Pilkington GJ (1995) Synergism between growth factors in the control of glioma cell proliferation, migration and invasion in vitro. *Int J Oncol* **6**: 1079–1085

Nakano A, Tani E, Miyazaki K, Furuyama J and Matsumoto T (1993) Expression of matrilysin and stromelysin in human glioma cells. *Biochem Res Commun* **192**: 999–1003

Nicolson GL, Nakajima M, Herrmann JL, Menter DG, Cavanaugh PG, Park SJ and Marchetti D (1994) Malignant melanoma metastasis to brain: role of degradative enzymes and responses to paracrine growth factors. *J Neuro-Oncol* **18**: 139–149

Overall CM, Wrana JL and Sodek J (1991) Transcriptional regulation of 72-kDa gelatinase/type IV collagenase by transforming growth factor β-1 in human fibroblasts. *J Biol Chem* **226**: 14064–14071

Pedersen P-H, Ness GO, Engebraaten O, Bjerkvig R, Lillehaug JR and Laerum OD (1994) Heterogeneous response to the growth factors [EGF, PDGF (bb), TGF-α, bFGF, IL-2] on glioma spheriod growth, migration and invasion. *Int J Cancer* **56**: 255–261

Pilkington GJ, Dunan JR, Rogers JP, Clarke TM and Knott JCA (1993) Growth factor modulation of surface ganglioside expression in cloned neoplastic glia. *Neurosci Lett* **149**: 1–5

Rao JS, Steck PA, Tofilon P, Boyd D, Ali-Osmon F, Stetler-Stevenson WG, Liotta LA and Sawaya R (1994) Role of plasminogen activator and 92-kDa type IV collagenase in glioblastoma invasion using an in vitro matrigel model. *J Neuro-Oncol* **18**: 129–138

Reubi JC, Horisberger U, Lang W, Koper JW, Braakman R and Lamberts SW (1989) Coincidence of EGF receptors and somatostatin receptors in meningiomas but inverse, differentiation-dependent relationship in glial tumors. *Am J Pathol* **134**: 337–344

Romanic AM and Madri JA (1994) Extracellular matrix-degrading proteinases in the nervous system. *Brain Pathol* **4**: 145–156

Rooprai HK and McCormick D (1997) Proteases and their inhibitors in human brain tumours: a review. *Anticancer Res* **17**: 4152–4162

Rooprai HK, Van Meter T, Rucklidge GJ, Hudson L, Everall IP and Pilkington GJ (1998) Comparative analysis of matrix metalloproteinases by immunocytochemistry, immunohistochemistry and zymography in human primary brain tumours. *Int J Oncol* **13**: 1153–1157

Rossi M, Rooprai HK, Maidment SL, Rucklidge GJ and Pilkington GJ (1996) The influence of sequential, in vitro passage on secretion of matrix metalloproteinases by human brain tumour cells. *Anticancer Res* **16**: 121–128

Rucklidge GJ, Lund-Johansen M, Milne G and Bjerkvig R (1990) Isolation and characterization of a metalloproteinase secreted by rat glioma cells in serum-free culture. *Biochem Biophys Res Commun* **172**: 544–550

Russell DS and Rubenstein LJ (1989) *Pathology of Tumours of the Nervous System*, 5th edn, pp. 83–289, Williams & Wilkins: Baltimore

Salo T, Lyons JG, Rahematulla F, Birkendal-Hansen H and Larjava H (1991) Transforming growth factor β-1 upregulates type IV collagenase expression in cultured human keratinocytes. *J Biol Chem* **226**: 11436–11441

Stetler-Stevenson WG, Brown PD, Onisto M, Levy AT and Liotta LA (1990) Tissue inhibitor of metalloproteinase-2 (TIMP-2) mRNA expression in tumour cell lines and human tumour tissues. *J Biol Chem* **265**: 13933–13938

Tamaki M, McDonald W and Del Maestro RF (1998) The importance of cell density in the interpretation of growth factor effects on collagenase IV activity release and extracellular matrix production from C6 astrocytoma cells. *J Neuro-Oncol* **39**: 205–216

Woessner JF (1991) Matrix metalloproteinases and their inhibitors in connective tissue remodeling. *FASEB* **5**: 2145–2154

Yamada KM (1983) Cell surface interactions with extracellular material. *Ann Rev Biochem* **52**: 761–799

© 2000 Cancer Research Campaign
